# Short- and long-term reliability of adult recall of vegetarian dietary patterns in the Adventist Health Study-2 (AHS-2)

**DOI:** 10.1017/jns.2014.67

**Published:** 2015-04-01

**Authors:** Marcia C. Teixeira Martins, Karen Jaceldo-Siegl, Jing Fan, Pramil Singh, Gary E. Fraser

**Affiliations:** 1 Department of Nutrition, Adventist University of São Paulo, Estrada de Itapecerica 5859, Jardim IAE, São Paulo, Brazil05858-001; 2 Department of Nutrition, School of Public Health, Loma Linda University, 24951 North Circle Drive, NH 1102, Loma Linda, CA 92350, USA; 3 Department of Epidemiology, School of Public Health, Loma Linda University, 24951 North Circle Drive, NH 2005, Loma Linda, CA 92350, USA

**Keywords:** Long-term reliability, Vegetarian diets, Dietary patterns, Adventists, Dietary recall, AHS-1, Adventist Health Study-1, AHS-2, Adventist Health Study-2, HHF, hospitalisation history form, HHF-3, third hospitalisation history form, PPV, positive predictive value, 5-RAS, short-term/5-year recall ability study, 33-RAS, long-term/33-year recall ability study, S-Stab, score of stability

## Abstract

Past dietary patterns may be more important than recent dietary patterns in the aetiology of chronic diseases because of the long latency in their development. We developed an instrument to recall vegetarian dietary patterns during the lifetime and examined its reliability of recall over 5·3 and 32·6 years on average. The short-term/5-year recall ability study (5-RAS) was done using 24 690 participants from the cohort of the Adventist Health Study-2 (mean age 62·2 years). The long-term/33-year recall ability study (33-RAS) included an overlap population of 1721 individuals who joined the Adventist Health Study-1 and Adventist Health Study-2 (mean age 72·5 years). Spearman correlation coefficients for recall of vegetarian status were 0·78 and 0·72 for the 5-RAS and 33-RAS, respectively, when compared with ‘reference’ data. For both time periods sensitivity and positive predictive values were highest for the lacto-ovo-vegetarian and non-vegetarian patterns (vegans, lacto-ovo-vegetarians, pesco-vegetarians, semi-vegetarians and non-vegetarians). In the 5-RAS analyses, male, non-black, younger, and more educated participants, lifetime Adventists, and those with more stability of consumption of animal products generally showed higher recall ability. Somewhat similar tendencies were shown for the 33-RAS analyses. Our findings show that the instrument has higher reliability for recalled lacto-ovo-vegetarian and non-vegetarian than for vegan, semi- and pesco-vegetarian dietary patterns in both short- and long-term recalls. This is in part because these last dietary patterns were greatly contaminated by recalls that correctly would have belonged in the adjoining category that consumed more animal products.

There is increasing evidence that non-communicable disease risks begin in fetal life and continue through to old age^(^^1^^–^^3^^)^. Adult non-communicable disease reflects cumulative lifetime exposures due to damaging physical and social environments. Diet plays an important role in this scenario. It is known that early consumption can influence disease risk later in life^(^^4^^,^^5^^)^. Moreover, dietary patterns change significantly with advancing age and estimates of recent diet by older individuals may not reflect the diet consumed over most of their adult life. Individuals who were diagnosed with a chronic disease may be particularly prone to change their diet; thus, their present dietary pattern may reflect past diet poorly. Therefore, a life-course approach to dietary patterns may be more important than an exclusive focus on recent diet when exploring the relationship of diet to risk of non-communicable diseases^(^^1^^)^.

To date, prospective studies of the health effects of the vegetarian diet commonly practised by Adventists have focused on current consumption of animal products (mainly red meat, poultry, fish, dairy products and eggs) reported at baseline. These studies have shown protective effects of vegetarian diets, as they are associated with lower risk of many non-communicable diseases^(^^6^^)^. Furthermore, the duration of adherence to a vegetarian diet may be relevant when predicting disease and mortality^(^^7^^–^^11^^)^.

Therefore, the reliability of recall of vegetarian dietary patterns is of considerable interest when relating these dietary patterns to risk of diseases, such as diabetes, CVD, hypertension and cancer, among others. To investigate this, we developed an instrument to recall vegetarian dietary patterns during the lifetime and examined its reliability for recall over short- and long-term periods among older adults.

## Subjects and methods

### Study population

The Adventist Health Study-2 (AHS-2) is a prospective cohort study of 96 335 members of the Seventh-day Adventist Church, in the USA and Canada. The design of the AHS-2 has been described in detail elsewhere^(^^12^^)^. Briefly, adult men and women of diverse ethnicity (mostly Caucasian or black/African-American), and aged ≥30 years were enrolled between 2002 and 2007. Participants completed a baseline questionnaire which included sections on diet, demographics, height, weight and lifestyle practices, and later, bi-annual hospitalisation history forms (HHF) administered every 2 years after the baseline questionnaire. A Californian subgroup of this population (*n* 1721) also participated in the Adventist Health Study-1 (AHS-1), conducted earlier (recruitment in 1976–1978)^(^^6^^,^^13^^)^. The present study was conducted according to the guidelines laid down in the Declaration of Helsinki and all procedures involving human subjects were approved by the Loma Linda University Institutional Review Board. Written informed consent was obtained from all subjects.

### Instrument to recall vegetarian dietary patterns

**Fig. 1.**
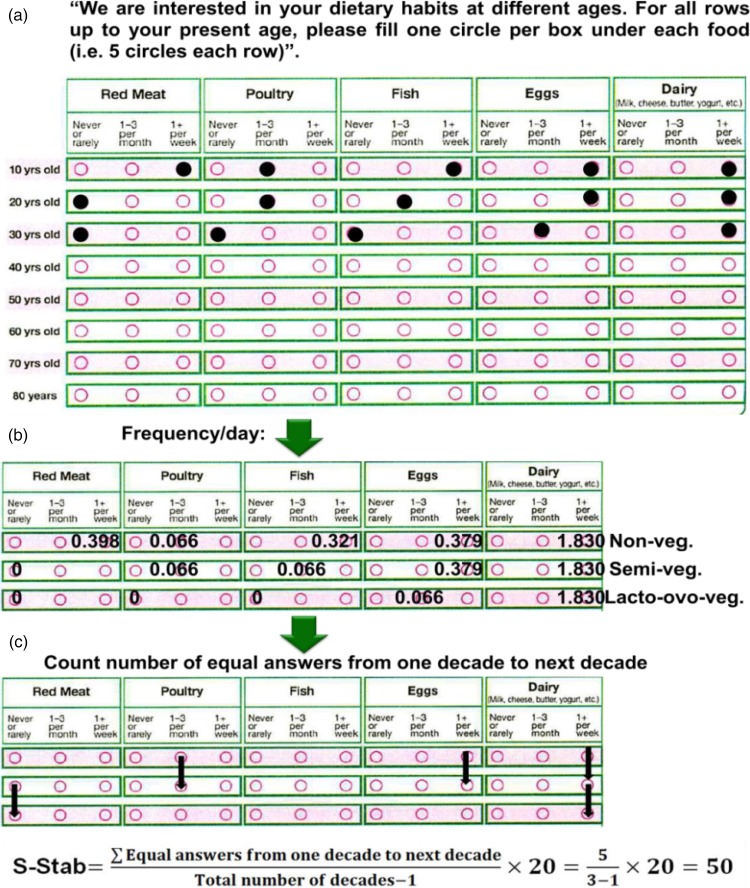
(a) Lifetime dietary habits instrument with an example of responses for an individual aged between 30 and 39 years (yrs). (b) An example of the corresponding vegetarian (veg.) dietary pattern classification according to consumption of animal products by decade. Numeral values represent daily weighted frequencies. (c) An example of the corresponding calculation of the score of stability (S-Stab) in the pattern of consumption of animal products.

The third HHF (HHF-3) was mailed from January 2009 to May 2010, and included a question that queried participants about their lifelong dietary habits: ‘We are interested in your dietary habits at different ages. For all rows up to your present age, please fill one circle per box under each food (i.e. 5 circles each row)’. The participants were required to recall their consumption of the five foods (the columns of the response table) needed to classify their vegetarian dietary pattern. These are also foods which in previous work tended to have better short- and long-term recall ability or validity than other foods^(^^14^^–^^16^^)^: meat, poultry, fish, eggs and dairy products. For each food subjects choose one of the three frequency options (never or rarely, 1–3 per month or ≥1 per week) for each decade from 10 years of age to their present age, up to a maximum age of 80 years (the rows of the response table). Fig. 1(a) shows an example of response to this question.

Among the AHS-2 participants, 63 919 completed the HHF-3. Since the purpose of this analysis was to evaluate the reliability of recall of vegetarian patterns, we excluded 20 % of participants who had more than one missing item on the lifelong dietary habits instrument; thus 51 082 were included in the analytical population. They were younger (61·9 (sd 12·9) *v.* 67·3 (sd 12·9) years), less likely to be female (64·0 *v.* 66·1 %), less likely to be black (16·4 *v.* 27·3 %) and had attained a higher educational level (44·9 *v.* 32·4 % college graduates) than subjects excluded. Some patterns of missing data for this question were identified, the more frequent being: one entire row (15 %), which corresponds to one decade of life or one entire column (2 %), corresponding to one food category. The 1534 cases with only one missing response were filled by multiple imputation^(^^17^^)^, conditional on the variables age, race, sex and education.

### Classification of vegetarian dietary patterns for each decade of life

Vegetarian dietary patterns are determined in the AHS-2 according to the frequency of intake of five animal foods (red meat, poultry, fish, eggs and dairy foods) obtained from a validated FFQ^(^^18^^,^^19^^)^. From these foods, three composite variables were then created by summing their reported intake frequencies: (1) meat (red meat + poultry); (2) dairy products/eggs (dairy products + eggs); and (3) fish as a separate group. Thus, vegans are participants who reported consuming meat, fish and dairy products <1 time per month. Lacto-ovo-vegetarians are those who consume dairy products ≥1 time per month, and fish and meat <1 time per month. Pesco-vegetarians are those who consume fish ≥1 time per month and meat <1 time per month. Semi-vegetarians are defined as consuming meat ≥1 time per month and the sum of meat and fish <1 time per week. Non-vegetarians are defined as those consuming meat ≥1 time per week. There were no restrictions on dairy product or egg intake for pesco-, semi- and non-vegetarians. Using these definitions for the HHF questions across all decades, we were able to classify subjects according to vegetarian dietary pattern for each decade of life. Frequencies of intake were converted to daily equivalents, as follows: never or rarely = zero/d, 1–3 per month ≈ 2 per month ≈ 0·066/d. Since the AHS-2 FFQ had five frequency options corresponding to 1+ per week in the HHF-3, this open-ended frequency category was estimated separately for each food to allow estimations that more closely represented the frequency choices of the present study population. We used the 1745 overlapping subjects who answered AHS-1, AHS-2 and the lifelong instrument to estimate the average frequencies/d for the 1+ per week category. Thus, for red meat, poultry and fish, we used 0·398/d, 0·383/d and 0·321/d, respectively. Fig. 1(b) shows the classification of vegetarian dietary patterns by decade of lifespan using the example of Fig. 1(a) and estimated frequencies per d.

### Assessment of reliability

Reliability of the vegetarian diet pattern classification as measured by the lifelong dietary habits instrument was assessed for both recent (short-term) and remote (long-term) recall. In the short-term recall reliability study (5-RAS), the mean elapsed time was 5·3 years (range 1·1–8·9 years) between the first measurement of diet at AHS-2 enrollment (2002–2007) and the second assessment – application of the HHF-3 (2009–2010). In the long-term recall reliability study (33-RAS), the mean elapsed time was 32·6 years (range 31–34 years) between report of diet at AHS-1 (1976–1978) and application of the HHF-3. Additional criteria for selection of these samples were applied and are described below.

Both studies included only individuals who at enrollment (AHS-1 and/or AHS-2) were aged ±2 years from the decade year stated in the HHF-3 assessment. For example, to assess recalled diet at decade 40 by the HHF-3, we chose subjects who were age 38–42 years during the initial assessments at baseline AHS-1 or AHS-2. For decade 50, we included subjects who were age 48–52 years, and so on. Decades 30 and 80 were exceptions. For these, we used subjects who were aged 30–32 years and 78–80 years at the initial assessment. With these criteria for selection, the final samples of cohort members available for the studies of short- and long-term of recall reliability were 24 690 and 1721 individuals, respectively. Thus, the analytic cohort used to explore long-term recall reliability of vegetarian dietary patterns was comprised of subjects who were participants in both studies (AHS-1 and AHS-2) and who also completed the HHF-3 of the AHS-2 study.

### Score of stability in the pattern of consumption of animal products

Lindsted & Kuzma^(^^14^^)^ created a ‘diet stability score’ to evaluate changes in the subjects' dietary patterns. They used this score as a determinant of the diet recall ability over 8 and 24 years^(^^20^^)^. We adapted this concept to the HHF-3 question about lifetime dietary habits and developed a score of stability (S-Stab) of consumption of animal products during the lifetime. Fig. 1(c) shows a calculation of the S-Stab applied to the example of Fig. 1(a) and (b). Possible S-Stab range from 0 to 100. Categories of stability of consumption of animal products were defined based on the median value (82) of their distribution among the individuals who participated in the 5-RAS. Individuals with S-Stab ≤82 were considered to have a less stable pattern of consumption of animal products and individuals with S-Stab >82 showed a more stable profile.

### Demographic and lifestyle exposures

Demographic and lifestyle data were obtained from the AHS-2 baseline questionnaire. Race and ethnicity were divided into black (African, American, West Indian/Caribbean, African American, or other black) and non-black (white, non-Hispanic, Hispanic, Middle Eastern, Asian, Native Hawaiian/other Pacific Islander or American Indian). Educational level was categorised as < college and ≥ college. Alcohol intake was defined as current consumption of any amount (yes/no), tobacco use was defined as current, past smoking (any amount), or never smoking, and current coffee consumption was scored as some or none. Typically, current smoking, or consumption of alcohol in Adventists, is uncommon. Coffee, when consumed, is usually one or two cups, or less, per d. Participants were also considered as lifetime Adventists if they were currently Adventists, also at the age of 15–25 years, and that either the mother or father who raised him/her was also Adventist when the participant was 0–15 years of age.

### Statistical methods

Analyses were performed using SAS, version 9.3 (SAS Institute Inc.). Frequencies, means, percentages, standard deviations and correlations were conducted as standard descriptive statistics. To assess reliability of recalled vegetarian dietary patterns between the HHF-3 and AHS-2 (5-RAS) and the HHF-3 and AHS-1 (33-RAS) we calculated Spearman rank-order correlation coefficients, sensitivity and positive predictive values (PPV). The initial measures (reference values) of the 33- and 5-RAS were AHS-1 and AHS-2 baseline FFQ, respectively. For the calculation of Spearman coefficients, diet patterns were ranked in order of content of animal products (i.e. vegan, lacto-ovo-, pesco-, semi- and non-vegetarian). Our software allowed adjustment for only one covariate. Thus, we report the range of Spearman values adjusting for each covariate separately. Logistic regressions were used for testing the significance of sensitivity and PPV for each dietary pattern by strata of demographic factors (sex, age, race, education, duration of church membership) and by the S-Stab in the pattern of consumption of animal products. For this purpose sensitivity and PPV are defined respectively for dietary pattern j as pr(HHF-3 = j|Reference = j) and pr(Reference = j|HHF-3 = j). All variables (except the stratification variables in Tables 3 and 4) were entered simultaneously into the logistic regression models. Age was dichotomised as ≤60 years and >60 years. Overall agreement is defined as the proportion that agreed exactly on recall with the original classification.

## Results

The 24 690 subjects in the 5-RAS represented the total AHS-2 cohort very well, both with respect to demographic variables and vegetarian dietary patterns (Table 1). As expected, however, the 1721 participants of the 33-RAS were older. Their mean age (72·5 years) was 10 years higher than that of the 5-RAS (62·2 years). Since this is a subgroup of participants of the AHS-1, a study designed mainly to study only white non-Hispanic California Adventists, there were very few black subjects (Table 1). As compared with 5-RAS participants, 33-RAS participants were white, better educated, reported a higher proportion with never use of alcohol and tobacco, were more likely to be current coffee consumers and to be lifelong Seventh-day Adventists, and had generally higher scores of stability of consumption of animal products during the lifespan. This group also showed higher proportions of lacto-ovo-vegetarians and semi-vegetarians, and lower proportion of vegans, pesco-vegetarians and non-vegetarians.

**Table 1.** Selected characteristics of participants in the Adventist Health Study-2 (AHS-2) cohort who were also part of the 5-year and 33-year dietary recall studies (Percentages or mean values and standard deviations)

	Total cohort	5-year	33-year
Participants (*n*)	51 082	24 690	1721
Sex (%)			
Males	36·0	36·2	37·4
Females	64·0	63·8	62·6
Age at HHF-3 (%)			
≤60 years	47·1	46·5	5·0
>60 years	52·9	53·5	95·0
Age (years)			
Mean	61·9	62·2	72·5
sd	12·9	12·8	8·4
Race (%)			
Black	16·4	16·0	0·2
Non-black	83·6	84·0	99·8
Education (%)			
Some college, high school or less	55·2	55·3	47·5
College or higher	44·8	44·7	52·5
Alcohol history (%)			
Never	59·8	59·7	75·2
Past	32·7	32·8	17·5
Current	7·5	7·5	7·3
Cigarette smoking (%)			
Never	82·5	82·7	91·2
Past	16·8	16·7	8·6
Current	0·7	0·6	0·2
Coffee, current (%)			
No	65·8	65·7	61·5
Yes	34·2	34·3	38·5
Lifetime SDA (%)			
No	45·1	44·7	27·1
Yes	54·9	55·3	72·9
Diet patterns at AHS-2 baseline (%)			
Vegan	8·6	8·5	6·9
Lacto-ovo-vegetarian	33·3	33·0	41·7
Pesco-vegetarian	9·4	9·4	8·4
Semi-vegetarian	5·7	5·6	7·0
Non-vegetarian	43·0	43·5	36·0
BMI (kg/m^2^)			
Mean	26·8	26·8	26·5
sd	5·8	5·8	5·0
Score of stability of consumption of animal products (%)			
≤82 (the median)	50·3	50·0	34·2
>82	49·7	50·0	65·8

HHF-3, hospitalisation history form 3; SDA, Seventh-day Adventist.

Table 2 presents overall (without covariate adjustment) recall reliability as estimated by Spearman's correlations and reliability of assignment of individual patterns as estimated by sensitivity and PPV in the 5-RAS and 33-RAS. As expected, the 33-RAS showed lower overall reliability than the 5-RAS, but Spearman's correlation coefficients from both studies were above 0·70 (0·78 and 0·72 for the 5-RAS and 33-RAS, respectively). In both studies sensitivity and PPV were higher for lacto-ovo-vegetarian and non-vegetarian dietary patterns, while semi-vegetarian and pesco-vegetarian patterns had poorer reliability. The vegan dietary pattern had comparatively better sensitivity in the 5-RAS, but lower sensitivity and PPV in the 33-RAS. Except for recalled lacto-ovo-vegetarian and non-vegetarian dietary patterns, sensitivity and PPV tended to decrease from 5 to 33 years of recall.

**Table 2.** Overall reliability of the vegetarian dietary patterns over 5-year and 33-year dietary recall periods, as measured by sensitivity, positive predictive value (PPV) and Spearmans ρ correlation coefficients

	5-year			33-year			Spearman's ρ*	
Vegetarian dietary pattern	AHS-2 (*n*)†	Sensitivity (%)	PPV (%)	AHS-1 (*n*)†	Sensitivity (%)	PPV (%)	5-year	33-year
Vegan	2103	75	42	18	50	16	0·78	0·72
Lacto-ovo-vegetarian	8150	71	77	818	81	81		
Pesco-vegetarian	2316	42	49	25	28	16		
Semi-vegetarian	1393	41	19	310	25	37		
Non-vegetarian	10 728	70	90	550	77	71		
Total	24 690			1721				

AHS-2, Adventist Health Study-2; AHS-1, Adventist Health Study-1.

* Spearman's correlation where the dietary patterns were ranked in the order vegan, lacto-ovo-vegetarian, pesco-vegetarian, semi-vegetarian and non-vegetarian.

† Number of individuals with survey response.

Table 3 shows that the overall 5-year reliabilities of recalled dietary patterns were moderately, but often significantly, lower among blacks, older and less educated individuals, and higher among lifetime Adventists, but with some variations between dietary patterns. An exception is the higher sensitivity among black pesco-vegetarians compared with white pesco-vegetarians. Sensitivity was usually higher among vegans, lacto-ovo-vegetarians and non-vegetarians compared with other vegetarian patterns.

**Table 3.** Reliability of adult recall of classification of vegetarian dietary patterns in the 5-year recall ability study by demographic and lifestyle factors and stability of consumption of animal products†

	Sensitivity (%)		Positive predictive value (%)		Overall agreement (%)	
By sex	Males (*n* 8945)	Females (*n* 15 745)	Males	Females	Males	Females
Vegan	68	66	51	49	68	66
Lacto-ovo-vegetarian	68	70	74	72		
Pesco-vegetarian	42	39	53	50		
Semi-vegetarian	42	39	19	18		
Non-vegetarian	71*	68	88	89		
Spearman's ρ‡	0·76–0·79	0·75–0·78				
By race	Non-blacks (*n* 20 732)	Blacks (*n* 3958)	Non-blacks	Blacks	Non-blacks	Blacks
Vegan	68	62	51*	41	67	64
Lacto-ovo-vegetarian	71*	60	74*	66		
Pesco-vegetarian	39*	49	51	52		
Semi-vegetarian	42*	32	21*	10		
Non-vegetarian	69*	73	88*	90		
Spearman's ρ‡	0·75–0·79	0·70–0·73				
By age§	Older (*n* 13 210)	Younger (*n* 11 480)	Older	Younger	Older	Younger
Vegan	71*	62	45*	55	64	70
Lacto-ovo-vegetarian	63*	75	68*	78		
Pesco-vegetarian	39	41	50	52		
Semi-vegetarian	38	43	17	20		
Non-vegetarian	63*	76	88*	89		
Spearman's ρ‡	0·74–0·78	0·78–0·80				
By education§	Less educated (*n* 13 645)	More educated (*n* 11 045)	Less educated	More educated	Less educated	More educated
Vegan	65	69	49	49	65	68
Lacto-ovo-vegetarian	67*	72	70*	77		
Pesco-vegetarian	38*	43	49	53		
Semi-vegetarian	40	40	18	20		
Non-vegetarian	70	69	88	89		
Spearman's ρ‡	0·74–0·77	0·77–0·80				
By lifetime SDA§	Non-lifetime SDA (*n* 11 049)	Lifetime SDA (*n* 13 641)	Non-lifetime SDA	Lifetime SDA	Non-lifetime SDA	Lifetime SDA
Vegan	71*	63	49*	44	65	68
Lacto-ovo-vegetarian	67*	71	71*	75		
Pesco-vegetarian	39	41	49	52		
Semi-vegetarian	37	42	16*	21		
Non-vegetarian	70	69	89	88		
Spearman's ρ‡	0·73–0·76	0·76–0·79				
By stability score§	≤82 (*n* 12 343)	>82 (*n* 12 347)	≤82	>82	≤82	>82
Vegan	73*	61	44*	49	57	76
Lacto-ovo-vegetarian	55*	80	63*	81		
Pesco-vegetarian	44*	37	47*	55		
Semi-vegetarian	38	42	18	19		
Non-vegetarian	56*	80	85*	91		
Spearman's ρ‡	0·69–0·73	0·83–0·85				

SDA, Seventh-day Adventist.

* *P* < 0·05. The *P* value tests the hypothesis that sensitivity and positive predictive value differ for different values of stratification variables.

†Sex, race, age, education, lifetime SDA, stability, alcohol, smoking and caffeinated coffee were entered simultaneously into the logistic regression model used for testing the significance of sensitivity and positive predictive value for each stratification variable. This was done separately for each of the five dietary patterns. Predicted values are estimated conditional on expected values for all variables except the variable that is the subject of the contrast.

‡ For each main stratification variable, Spearman correlation coefficients (ρ) were adjusted by each covariate separately and the range of results is reported.

§ Older and younger individuals were defined as those with age >60 years and ≤60 years of age, respectively; less and more educated individuals were categorised as some college, high school or less and college and above, respectively; lifetime SDA participants were SDA at the age of 15–25 years and either the mother of father who raised him/her was also Adventist when the participant was 0–15 years of age; non-lifetime SDA is the opposite of lifelong SDA; less and more stable individuals were those who presented values of the score of stability of consumption of animal products ≤82 and >82, respectively.

Somewhat similar tendencies were observed for the 33-RAS (Table 4), but with few statistically significant results when comparing between subjects in the different categories of that stratification variable. Overall agreement tended to be lower among less educated, non-lifetime Adventists and those with lower stability scores. The low number of black and younger participants limited the precision of comparisons by age and race categories, which are not shown.

**Table 4.** Reliability of adult recall of classification of vegetarian dietary patterns in the 33-year recall ability study by demographic and lifestyle factors and stability of consumption of animal products†

	Sensitivity (%)		Positive predictive value (%)		Overall agreement	
By gender	Males (*n* 643)	Females (*n* 1078)	Males	Females	Males	Females
Vegan	–	–	19	29	69	68
Lacto-ovo-vegetarian	78	79	79	79		
Pesco-vegetarian	–	–	–	–		
Semi-vegetarian	28	21	47	35		
Non-vegetarian	75	76	71	71		
Spearman's ρ‡	0·69–0·73	0·69–0·72				
By education§	Less educated (*n* 818)	More educated (*n* 903)	Less educated	More educated	Less educated	More educated
Vegan	–	–	35	17	67	70
Lacto-ovo-vegetarian	76	81	78	79		
Pesco-vegetarian	–	–	–	–		
Semi-vegetarian	24	23	37	42		
Non-vegetarian	77	74	71	72		
Spearman's ρ‡	0·67–0·70	0·67–0·72				
By lifetime SDA§	Non-lifetime SDA (*n* 466)	Lifetime SDA (*n* 1255)	Non-lifetime SDA	Lifetime SDA	Non-lifetime SDA	Lifetime SDA
Vegan	–	–	23	26	65	70
Lacto-ovo-vegetarian	78	79	75	80		
Pesco-vegetarian	–	–	–	–		
Semi-vegetarian	22	25	36	41		
Non-vegetarian	72	77	74	70		
Spearman's ρ‡	0·65–0·69	0·70–0·73				
By stability score§	≤82 (*n* 588)	>82 (*n* 1133)	≤82	>82	≤82	>82
Vegan	–	–	22	26	61	72
Lacto-ovo-vegetarian	67*	83	72*	81		
Pesco-vegetarian	–	–	–	–		
Semi-vegetarian	28	22	45	37		
Non-vegetarian	72	77	63*	75		
Spearman's ρ‡	0·55–0·58	0·75–0·79				

–, Too few subjects in this category (estimates unstable); SDA, Seventh-day Adventist.

* *P* < 0·05. The *P* value tests the hypothesis that sensitivity and positive predictive value differ for different values of stratification variables.

†Sex, age, education, lifetime SDA, stability, alcohol, smoking and caffeinated coffee were entered simultaneously into the logistic regression model used for testing the significance of sensitivity and positive predictive value for each stratification variable. This was done separately for each of the five dietary patterns. Coffee could not be included for vegan predicted value positive results as there were too few coffee drinkers and estimates were then unstable. Predicted values are estimated conditional on expected values for all variables except the variable that is the subject of the contrast.

‡ For each main stratification variable, Spearman correlation coefficients (ρ) were adjusted by each covariate separately and the range of results is reported.

§ Less and more educated individuals were categorised as some college, high school or less and college and above, respectively; lifetime SDA participants were SDA at the age of 15–25 years and either the mother of father who raised him/her was also Adventist when the participant was 0–15 years of age; non-lifetime SDA is the opposite of lifelong SDA; less and more stable individuals were those who presented values of the score of stability of consumption of animal products ≤82 and >82, respectively.

It was also noticeable that PPV, and in some cases also sensitivity, tended to be higher among those who had higher S-Stab. This was especially true for lacto-ovo-vegetarians and for non-vegetarians. It seems that individuals with fewer changes in their pattern of consumption of animal products during their lifetime showed better recall ability of their past diet, over this longer period. Overall agreement generally showed similar trends to those reported above, and results were typically in the 64–70 % range with no clear differences between the 5- and 33-year follow-ups. Again the most striking loss of agreement was among those with lower dietary stability.

When subjects' recollections of their previous diet (using the HHF-3) were different from baseline data of either the 5-RAS or 33-RAS (data not shown), these misperceptions tended to reallocate subjects who were originally nearer to the closest adjoining category that consumed fewer animal products, into that adjoining category (Table 5).

**Table 5.** Adventist Health Study-2 (AHS-2) baseline intakes of meat, fish and dairy products/eggs by cross-tabulation of dietary patterns between AHS-2 baseline and later recall (5-year recall ability study; 5-RAS): frequencies of intake per d

					Recalled dietary pattern and actual intake at AHS-2 baseline																			
	Intake at AHS-2 baseline (all subjects)				Recalled as vegan				Recalled as lacto-ovo-vegetarian				Recalled as pesco-vegetarian				Recalled as semi-vegetarian				Recalled as non-vegetarian			
AHS-2 baseline dietary pattern	Meat	Fish	Dairy	*n*	Meat	Fish	Dairy	*n*	Meat	Fish	Dairy	*n*	Meat	Fish	Dairy	*n*	Meat	Fish	Dairy	*n*	Meat	Fish	Dairy	*n*
Vegan	0	0	0	2103	0	0	0	1583	0	0	0	399	0	0	0	48	0	0	0	32	0	0	0	43
Lacto-ovo-vegetarian	0	0	1·29	8150	0	0	0·58	1584	0	0	1·48	5760	0	0	1·21	264	0	0	1·57	321	0	0	1·30	221
Pesco-vegetarian	0	0·21	1·32	2316	0	0·18	0·69	299	0	0·13	1·58	519	0	0·26	1·36	979	0	0·15	1·43	229	0	0·25	1·31	290
Semi-vegetarian	0·09	0·02	1·61	1393	0·08	0·02	0·87	102	0·08	0·02	1·67	329	0·07	0·04	1·57	78	0·09	0·02	1·73	566	0·09	0·02	1·55	319
Non-vegetarian	0·59	0·23	2·11	10 728	0·34	0·21	1·31	239	0·33	0·15	2·02	516	0·32	0·31	1·72	611	0·38	0·17	2·0	1810	0·68	0·24	2·20	7553
Total				24 690				3807				7523				1980				2958				8426

Referring to Table 5, a total of 3807 participants recalled themselves as being vegans at their baseline age. Of these, 1583 (42 %) were true positives and 58 % were false positives. The majority of the false positives (71 %) were in fact originally classified as lacto-ovo-vegetarians. Interestingly, when we examined their baseline daily frequencies of dairy products and eggs we found that these misclassified individuals originally consumed lower amounts of dairy products and eggs (0·58/d) than all lacto-ovo-vegetarians (1·29/d).

Similarly, a total of 1980 individuals were classified as pesco-vegetarians in the HHF-3. Of these, 49 % were true positives and 51 % were false positives. Most of the false positives (61 %) were in fact originally non-vegetarians who at baseline reported higher average daily frequency of fish (0·31/d) and lower daily frequency of meat (0·32/d) than all non-vegetarians (0·23/d and 0·59/d, respectively).

The same pattern was also observed among the 2958 participants who were classified as semi-vegetarians in the HHF-3. Of these, only 19 % were true positives and 81 % were false positives. However, most of the false positives (76 %) were in fact originally non-vegetarians who had lower daily frequency of meat and fish than all non-vegetarians (0·38/d *v.* 0·59/d and 0·17/d *v.* 0·23/d, respectively).

A different pattern was found for those who recalled themselves as being lacto-ovo-vegetarians (*n* 7523). Among them 77 % were true positives and 23 % were false positives. Most of the false-positive cases were either pesco-vegetarians (7 %) who ate less fish (0·13/d) than all pesco-vegetarians (0·21/d) or non-vegetarians (7 %) who ate less meat (0·33/d) than all non-vegetarians (0·59/d). A total of 90 % of those who recalled themselves as being non-vegetarians (*n* 8426) were true positives.

As the original non-vegetarian and lacto-ovo-vegetarian categories are large, proportionately small erroneous transfers out of these categories on recall overwhelm the number of subjects who recall accurately in the smaller adjoining dietary categories. Thus, most of the error measured by PPV (false positives) was derived from this type of misclassification.

## Discussion

In this report we describe an instrument designed to recall vegetarian dietary patterns during the lifetime and evaluated the reliability of recall of dietary patterns over 5·3 and 32·6 years on average. We found that the instrument has higher reliability for recalled lacto-ovo-vegetarian and non-vegetarian dietary patterns compared with vegan, semi- and pesco-vegetarian dietary patterns in both short- and long-term recalls. In the 5-RAS, males, non-blacks, younger and more educated participants, lifetime Adventists, and those with higher stability of consumption of animal products had greater reliability. Somewhat similar tendencies were shown for the 33-RAS. The subjects misclassified by recall to the three less reliable categories (vegans, pesco- and semi-vegetarians) often tended to recall to the nearest ‘more ideal’ category, from a vegetarian perspective. Such misclassification might be explained arithmetically due to the limited consumption frequency categories available in the HHF-3 or may indicate social desirability bias.

The assessment of past diet, and its reliability and relative validity have been previously investigated^(^^3^^,^^16^^,^^21^^–^^30^^)^ with diet recalls from 1 year to 48 years later. As compared with current diet, others have found that the recalled diet usually provides a more reliable estimate of past diet, both for nutrients and food group variables. Five studies have previously investigated recall ability in Adventist populations^(^^14^^–^^16^^,^^20^^,^^31^^)^. The factors found to influence recall reliability include the duration of recall period, data collection methods, the respondents' demographic characteristics (sex, age, etc.) and the dietary factors involved^(^^22^^,^^25^^)^.

### Previous assessments of lifetime diet stability in Adventists

The present results extend the work of Lindsted & Kuzma^(^^14^^,^^15^^)^, who also studied an Adventist population and observed a statistically significant trend of decreasing recall reliability with increasing age. They investigated the determinants of reliability of short- (8 years)^(^^31^^)^ and long-term diet recalls (24 years)^(^^20^^)^ among participants of the Adventist Mortality Study. Unlike other studies, our instrument did not collect information about many food items relating to one single age in the past. Instead, we collected information of only five food items but covered all previous decades of life. This could have involved an additional challenge to our respondents, especially older ones, who had to recall a larger number of previous decades.

Studies have shown that recall ability is dependent on a number of factors related to diet, including the frequency of consumption of food items and the importance placed on them. Recall seems to be most reliable for foods eaten rarely^(^^22^^,^^23^^)^. Lindsted & Kuzma^(^^15^^)^ observed that the 8-year recall reliability was higher for food items considered as ‘important’ by Adventist traditions and recommendations, such as meat, beef, non-fat milk, low-fat milk, buttermilk and eggs. Similar results were found by the same authors for 24-year recall^(^^14^^)^. For example, foods that are often avoided, such as meat use in vegetarians, were recalled well. Fraser *et al.*^(^^16^^)^ found similar results when addressing the validity of 20-year dietary recall among Californian Adventists. Beef and chicken, and all meats combined, were recalled with validity coefficients of greater than 0·4. The highest coefficient was for hamburgers (0·55–0·88) based on the different methods of scoring the FFQ. Eggs, whole milk and fish had correlation coefficients of greater than 0·3. Again, these were foods observed to be ‘important’ to many Adventists.

In the present study, we asked only about foods used to classify vegetarian dietary patterns (red meat, poultry, fish, eggs and dairy products). Many Adventists have a special interest in diet and will long ago have made decisions regarding these foods. This fact may have helped the participants of the present study to remember their previous consumption of these food items. For this population, consumption of animal products probably became components of the so-called ‘permanent diet memory’ that reflects the basal consumption rate of ‘usual diet’ and includes category labels, such as: ‘foods I eat’ or ‘foods I never eat’. Memories of ‘usual’ dietary habits are stored as permanent and their report has been described as independent of time^(^^32^^)^. This permanent diet memory can be both a strength and a weakness in the analysis of dietary intake. It is a weakness in that it drives social desirability bias, which probably accounts for the results of Table 5 where many subjects tend to recall fewer animal products than they actually consumed. If animal products have an adverse effect on health, this may somewhat exaggerate the apparent effects of these foods. On the other hand, for individuals who choose a diet for health or philosophical reasons, the permanent diet memory is an advantage as it helps them recall which foods they avoided, and should also improve recall ability. It is unclear whether these findings will apply to the general population.

Other demographic characteristics that may influence recall reliability were examined by Kuzma & Lindsted^(^^20^^,^^31^^)^. They found in their 8-year recall study that the best predictors of recall were diet stability, vegetarian status, higher education, higher income, and, as already mentioned, younger age^(^^31^^)^. In their 24-year recall study, the best predictors were also diet stability, vegetarian status, higher education and, to a lesser extent, regular church attendance^(^^20^^)^. Overall, the key determinant of diet recall was diet stability over time. Accordingly, highest correlation coefficients were observed for subjects who reported no dietary change^(^^22^^,^^23^^)^. We also found that lifetime Adventists with higher education who had had more stable dietary habits tend to perform better when recalling foods that determined their vegetarian dietary patterns.

Although many studies have explored agreement between recalled and original diet for nutrients and food groups, as far as we are aware, no other study has focused on the reliability of recall of vegetarian dietary patterns. The closest approach was the study of Eysteinsdottir *et al.*^(^^30^^)^ in which midlife diet was recalled 18 to 19 years later when subjects were categorised into five groups according to level of consumption of each food. Interestingly, cross-classification showed that 16–59 % were classified into the same group and 43–91 % into the same or adjacent group and 0–14 % were grossly misclassified. The same trend was observed in the present study when in both the 5-RAS and 33-RAS, the lifetime dietary habits instrument in the HHF-3, when incorrect, tended to classify subjects into the closest adjoining category that consumed fewer animal products. Probably this is an example of social desirability bias in this population. In the present paper we chose to focus on the recall of vegetarian dietary patterns because these patterns, although defined by few foods, have large differences in consumption of many nutrients, vitamins and minerals^(^^33^^)^. Moreover, they are associated with differences in many disease outcomes and total mortality^(^^6^^,^^34^^)^. For this reason, some believe that dietary pattern analysis is more useful in predicting disease risk than individual foods or nutrients^(^^35^^–^^37^^)^.

### Duration of recall period and reliability

Others have reported that correlations between the original and recalled reports decrease as the recall time becomes longer^(^^22^^,^^23^^,^^29^^)^. This was also observed in the present results as the Spearman correlation coefficients, sensitivity and PPV tended to decrease from the 5-RAS to the 33-RAS.

Previous studies showed that, overall, sex does not appear to influence recall ability^(^^22^^,^^23^^)^. This was confirmed by the present work, although men had slightly higher correlations in both the 5-RAS and 33-RAS.

### Age and reliability of recall

Advanced age might be related to decreased recall ability to access past diet^(^^22^^)^. However, some studies did not show differences in recall reliability with increased age^(^^22^^,^^23^^)^. It seems that long-term memory often remains intact despite reductions in short-term memory. Therefore accuracy of the memory of foods consumed does not necessarily decline over time^(^^25^^,^^38^^)^. However, reliability of dietary assessment methods applied in older individuals is dependent on the group of elderly individuals under study and the type of information required^(^^39^^)^. To date, we know of only two other studies that have evaluated diet recall over more than 30 years^(^^25^^,^^29^^,^^40^^)^. They assessed recall of adolescent diet with better correlations after 32 years^(^^40^^)^ than after 48 years^(^^29^^)^. Although Dwyer *et al.*^(^^40^^)^ concluded that the recall of food intake in the distant past may be a sufficiently valid estimate of past intake, the conclusion by Chavarro *et al.*^(^^29^^)^ was that their instrument did not measure overall adolescent diet with adequate validity when completed by middle-aged and older adults. In the present study, recall of vegetarian dietary patterns from the distant past (33 years) had good long-term recall reliability for lacto-ovo-vegetarian and non-vegetarian patterns, while pesco-vegetarian and semi-vegetarian patterns had lower recall reliability. However, the lowest Spearman's correlation coefficient for the 33-RAS (thus across all dietary patterns) was 0·55 in the group with the lower stability score.

### Limitations

One of the limitations of the present study is that the exclusion criteria (participants who had more than one missing response on the lifelong dietary habits instrument) eliminated 20 % of the participants from the study. The individuals excluded were demographically different from the remaining participants. They were older, more likely to be black and women, and had a lower educational level on average. Thus it is also possible that this group may have poorer recall ability. A proportion of the remaining individuals left one missing response on the instrument (3 %; *n* 1534). We filled these blank answers by multiple imputation, a strategy that requires the assumption that these missing data are missing at random. This assumption is difficult to verify, but with such a small percentage of missing data, even moderate deviations from this assumption will not have much influence^(^^41^^)^.

The method of data collection is one of the factors influencing recall reliability. Previous studies showed lower correlations for studies that used different instruments for original and recalled diet assessments^(^^22^^)^. Therefore, an important limitation of the present study is that the instrument used as the referent measure is different from the short set of questions about lifelong dietary habits used in the HHF-3. For example, the latter focused on the composite intakes of five foods from animal sources whereas the referent measure was a more comprehensive FFQ which included multiple questions about each of the five foods. Intake of animal foods from the HHF-3 instrument may be underestimated relative to the more comprehensive food list from the referent FFQ. The effect could be misclassification of dietary patterns toward a more plant-based diet (which is what we observed), so attenuating the reliability measurements.

### Conclusion

To date, most studies of Adventist populations have used the classification of the vegetarian dietary patterns based on the recent consumption of animal products. However, some studies showed that the duration of adherence to a vegetarian diet is important when addressing mortality and risk of disease^(^^7^^–^^11^^)^. We conclude that our instrument to recall vegetarian dietary patterns is an inexpensive, useful tool with which to reliably classify lacto-ovo- and non-vegetarian dietary patterns across long periods of life in this population. The instrument does classify some subjects who originally claimed to belong in these patterns, but who consumed relatively fewer animal products, into the next ‘more vegetarian’ pattern. This has an adverse impact on the reliability of the less frequent dietary patterns.
